# Ten-Year Follow-Up of a Patient with Metastatic Ewing's Sarcoma of the Pelvis

**DOI:** 10.1080/1357714021000022140

**Published:** 2002-12

**Authors:** Robert U. Ashford, Eugene V. McCloskey, O. Prakash Purohit, Christine E. Ingram, Robert J. Grimer, Robert E. Coleman

**Affiliations:** 1 Sheffield Bone Metastases Multidisciplinary Team Sheffield Metabolic Bone Unit Royal Hallamshire Hospital Sheffield UK; 2 Sheffield Bone Metastases Multidisciplinary Team Department of Clinical Oncology Weston Park Hospital Sheffield UK; 3 Sheffield Bone Metastases Multidisciplinary Team Department of Radiology Royal Hallamshire & Weston Park Hospitals Sheffield UK; 4 Sheffield Bone Metastases Multidisciplinary Team Royal Orthopaedic Hospital Oncology Service Birmingham UK; 5 Sheffield Bone Metastases Multidisciplinary Team Department of Medical Oncology Weston Park Hospital Sheffield UK; 6 MRCS (Glasg.) Rose Cottage Crooked Lane Kirk Hammerton York YO26 8DG UK

## Abstract

*Patient:* We report a 32-year-old women with a pelvic Ewing's sarcoma, who developed skeletal metastases within 20 months of diagnosis but following treatment remains disease-free at 10 years.

*Discussion:* Ewing's sarcoma is a highly malignant tumour of bone. Presentation of a pelvic tumour over the age of 30 years is extremely rare and associated with a poor prognosis. Early recurrence is also associated with an extremely grave prognosis. In our patient there was little or no response to salvage chemotherapy, but, against the odds, remission was induced with pamidronate and palliative radiotherapy to some but not all sites of disease.This remission has been maintained without additional therapy for a further 5 years.


*Sarcoma* (2002) **6**, 131–133

Taylor & Francis healthsciences

**CASE REPORT**

### Ten-year follow-up of a patient with metastatic Ewing's sarcoma of the pelvis

ROBERT U. ASHFORD[1], EUGENE V. McCLOSKEY[1], O. PRAKASH PUROHIT[2], CHRISTINE E. INGRAM[3], ROBERT J. GRIMER[4] & ROBERT E. COLEMAN[5]

*Sheffield Bone Metastases Multidisciplinary Team, [1]Sheffield Metabolic Bone Unit, Royal Hallamshire Hospital, Sheffield, UK, [2]Department of Clinical Oncology, Weston Park Hospital, Sheffield, UK, [3]Department of Radiology, Royal Hallamshire & Weston Park Hospitals, Sheffield, UK, [4]Royal Orthopaedic Hospital Oncology Service, Birmingham, UK, [5]Department of Medical Oncology, Weston Park Hospital, Sheffield, UK,*

**Abstract**

*Patient:* We report a 32-year-old women with a pelvic Ewing's sarcoma, who developed skeletal metastases within 20 months of diagnosis but following treatment remains disease-free at 10 years.

*Discussion:* Ewing's sarcoma is a highly malignant tumour of bone. Presentation of a pelvic tumour over the age of 30 years is extremely rare and associated with a poor prognosis. Early recurrence is also associated with an extremely grave prognosis. In our patient there was little or no response to salvage chemotherapy, but, against the odds, remission was induced with pamidronate and palliative radiotherapy to some but not all sites of disease. This remission has been maintained without additional therapy for a further 5 years.

**Key words:** *Ewing's sarcoma, pelvis, bisphosphonates, metastases*

### Introduction

Metastatic Ewing's sarcoma of the pelvis has an extremely poor prognosis in adult patients.[1] We report the case of a woman aged 32 years at diagnosis, who developed skeletal metastases within 20 months of diagnosis but following treatment remains disease-free at 10 years.

### Case report

A 32-year-old woman was referred to Weston Park Hospital in 1990 with a Ewing's sarcoma of the right superior pubic ramus (Fig. 1a,b). Following three cycles of pre-operative chemotherapy with ifosfamide vincristine and doxorubicin (IVAD), she underwent a two-stage tumour excision and endoprosthesis with a titanium hemipelvic replacement. She then received post-operative chemotherapy with the same agents, substituting actinomycin D for doxorubicin after a total of six cycles of IVAD during which time her renal function deteriorated (Table 1). Her endoprosthetic surgery was complicated by a streptococcal wound infection and subsequently dislocation of her prosthesis.

Eight months after completion of chemotherapy (20 months after surgery), she experienced discomfort in the chest, and a chest radiograph revealed multiple lytic metastases in the ribs (Fig. 2). CT scanning demonstrated no soft tissue extension of these lesions. A 99-technetium isotope bone scan revealed areas of increased uptake in the pelvis, ribs, spine and sternum, consistent with multiple metastases (Fig. 3, left). Her full blood count was normal. Given the typical appearances of metastases both radiologically and on isotope bone scanning, confirmatory biopsy was not performed. She was subsequently treated with five cycles of carboplatin and methotrexate. A further cycle of chemotherapy was not given because of deterioration in her renal function as assessed by both creatinine clearance (Table 1) and by an increase in creatinine from 90 μmol/l (pre-chemotherapy) to 250 μmol/l. The chest pain persisted and required radiotherapy (single fraction of 600cGy) to the left lateral ribs and the left hip (2000 cGy in five fractions). In addition, three cycles of intravenous pamidronate (120 mg per cycle) were also given for the bone pain, with good, but temporary, symptomatic relief.

Correspondence to: Mr Robert U. Ashford MRCS (Glasg.), Rose Cottage, Crooked Lane, Kirk Hammerton, York YO26 8DG, UK. Tel.: +44-1423-339361; E-mail: robert.ashford@virgin.net

ISSN 1357–714X print/ISSN 1369–1643 online/02/0200131–04 © Taylor & Francis Ltd
DOI: 10.1080/1357714021000022140

**Table 1.** *Creatinine clearance in this patient over time*

| Date | Notes | Creatinine clearance (ml/min) |
|---|---|---|
| 1990 | Pre-chemotherapy | 90 |
| January 1991 | Post IVAD | 70 |
| December 1991 | After recurrence | 50 |
| Late 1992 | After salvage chemotherapy | 30 |
| All subsequent measurements | | 30–35 |

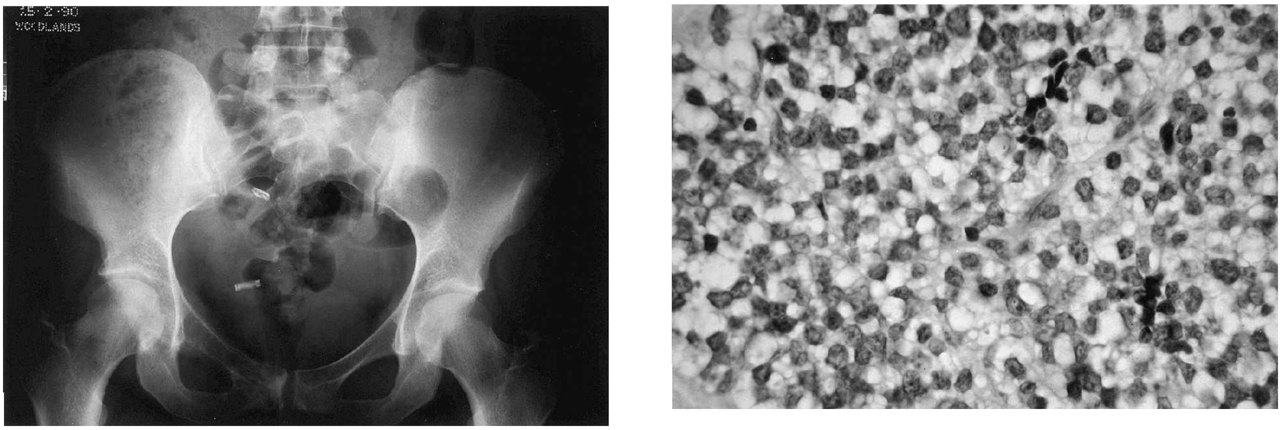

**Fig. 1.** *(a) Radiograph of the pelvis demonstrating a tumour of the right superior pubic ramus. (b) Histology (H&E) characteristic of Ewing's sarcoma.*

Six months later (late 1992), a further 6-week course of low-dose weekly palliative chemotherapy with vincristine, epirubicin and cyclophosphamide was administered. This did not produce any significant symptomatic improvement and was followed by palliative radiotherapy to the left shoulder (800 cGy in a single fraction) and again to the left hip (800 cGy in a single fraction). Intermittent infusions of pamidronate 120 mg were continued every 4 to 12 weeks as required for pain with good symptomatic improvement. A further course of fractionated radiotherapy (1250 cGy in five fractions) for a recurrence of severe bone pain in her lower ribs was required in late 1993. Isotope bone scanning at this stage demonstrated further increased uptake in the pubic rami and persistence of the other skeletal lesions.

In 1994, she suffered insufficiency fractures of both left pubic rami and underwent investigations of bone metabolism. These revealed normal calcium, vitamin D and parathyroid hormone but grossly elevated hydroxyproline and pyridinoline. A unicortical, double-labelled iliac crest bone biopsy revealed renal osteodystrophy with signs of hyperparathyroidism and no evidence of osteomalacia. Treatment with calcitriol corrected her abnormal bone biochemistry. Isotope bone scanning at that stage (1995) revealed no evidence of skeletal metastases, and the focal increased uptake in the ribs had disappeared (Fig. 3, right).

Since 1995, the patient has remained in remission with a recent assessment revealing no evidence of metastatic disease. Serial isotope bone scans remain unchanged from 1995.

### Discussion

Ewing's sarcoma is a highly malignant round-cell tumour of bone that occurs most commonly between the ages of 10 and 25 years. Presentation over the age of 30 years, as in our patient, is extremely rare and is associated with a poor prognosis.[1] The pelvis is the most commonly affected site[2] and carries an especially poor prognosis with high rates of both local recurrence and distant metastases.[1–4] Surgical resection in combination with chemotherapy has improved the prognosis of pelvic Ewing's sarcoma,[1–4] but the outlook remains poor in those patients who develop skeletal metastases. Of the seven patients over the age of 20 years managed at UCLA,[3] who developed bone

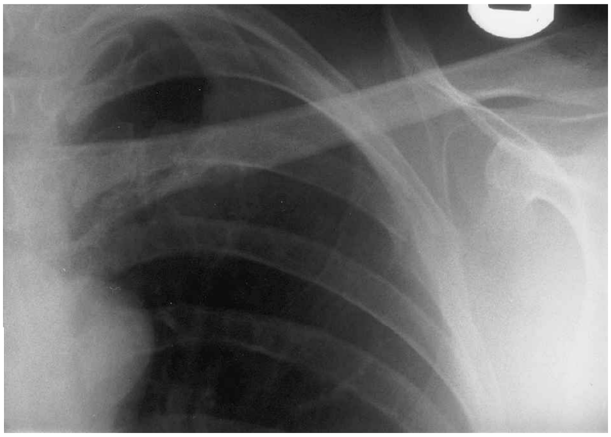

**Fig. 2.** *Radiograph of ribs demonstrating destructive osteolytic lesions affecting ribs 3 and 4.*

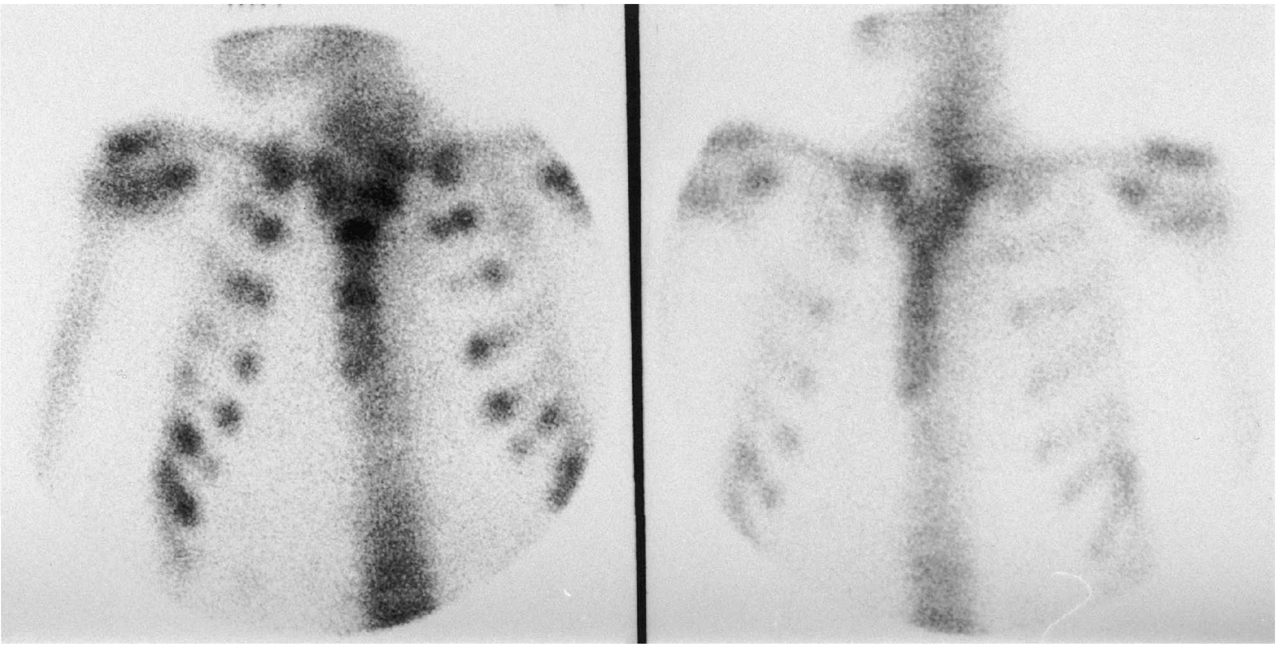

**Fig. 3.** *99-Tc isotope bone scans. (Left) pre-therapy with multiple metastases and (right) post-therapy with resolution and remission induced.*

metastases, the median survival from date of primary diagnosis was 21 months (range 6–56 months). Similarly, in two patients of the same age group in the Mayo Clinic,[4] death occurred within a year of diagnosis. Early recurrence can therefore be seen to be associated with an extremely grave prognosis.

This patient had widespread lytic skeletal metastases, which developed within 2 years of the primary tumour, treated by a combination of chemotherapy, radiotherapy and bisphosphonates. There was little or no response to salvage chemotherapy, but, against the odds, remission was induced with pamidronate and palliative radiotherapy to some but not all sites of disease. This remission has been maintained without additional therapy for a further 5 years. It is unlikely that radiotherapy played a major role against the disease given the disseminated nature of the skeletal changes on isotope bone scanning. It may, however, be that salvage chemotherapy in 1991 and 1992 was more effective than we estimated. What goes against this is that prolonged survival with salvage chemotherapy in metastatic Ewing's disease is extremely rare.

The major determinant of a lack of response in this case is persisting pain and poor mobility. However, follow-up X-rays failed to demonstrate healing of the bone lesions during chemotherapy. Additionally, bone scanning only began to show fading of the hot spots from 1995.

Bisphosphonates have potent effects on bone metabolism and the bone marrow microenvironment. There are also *in vitro* data that suggest that high concentrations of bisphosphonates may inhibit tumour cell proliferation and promote apoptosis of tumour cells.[5] Whether this remarkable response is related to these phenomena is impossible to conclude, but the possibility is intriguing and further *in vivo* evaluation of the 'antitumour effects' of bisphosphonates is required.

This is a complex case and no one intervention is responsible for the prolonged survival in this patient.

### Acknowledgements

The authors wish to thank Dr Charles Mangham, Department of Musculo-Skeletal Pathology, Selly Oak Hospital, Birmingham, for providing the histology slides.

### References

1. Baldini EH, Demetri GD, Fletcher CDM, Foran J, Marcus KC, Singer S. Adults with Ewing's sarcoma/ primitive neuroectodermal tumor: adverse effect of older age and primary extraosseous disease on outcome. *Ann Surg* 1999; 230(1): 79–86.
2. Hoffman C, Ahrens S, Dunst J, *et al.* Pelvic Ewing sarcoma: a retrospective analysis of 241 cases. *Cancer* 1999; 85(4): 869–77.
3. Yang R-S, Eckhardt JJ, Eilber FR, *et al.* Surgical indications for Ewing's sarcoma of the pelvis. *Cancer* 1995; 76(8): 1388–97.
4. Frassica FJ, Frassica DA, Pritchard DJ, Schomberg PJ, Wold LE, Sim FH. Ewing sarcoma of the pelvis. Clinicopathological features and treatment. *J Bone Jt Surg* 1993; 75A(10): 1457–65.
5. Jagdev S, Coleman RE, Shipman CM, Amin Rostami-H, Croucher PI. The bisphosphonate zoledronic acid induces apoptosis of breast cancer cells: evidence for synergy with paclitaxel. *Br J Cancer* 2001; 84(8): 1126–34.
